# Design and process evaluation of an informative website tailored to breast cancer survivors’ and intimate partners’ post-treatment care needs

**DOI:** 10.1186/1756-0500-5-548

**Published:** 2012-10-03

**Authors:** Evelyn Pauwels, Elke Van Hoof, Caroline Charlier, Lilian Lechner, Ilse De Bourdeaudhuij

**Affiliations:** 1 Faculty of Medicine and Health Sciences, Department of Movement and Sport Sciences, Ghent University, Watersportlaan 2, 9000, Gent, Belgium; 2 Faculty of Psychological and Educational Sciences, Free University of Brussels, Brussels, Belgium; 3 Belgian Cancer Centre, Scientific Institute of Public Health, Brussels, Belgium; 4 Faculty of Psychology, Open University of the Netherlands, Heerlen, the Netherlands

**Keywords:** Breast cancer, Partner, Design study, Process evaluation, Internet, Survivorship

## Abstract

**Background:**

On-line provision of information during the transition phase after treatment carries great promise in meeting shortcomings in post-treatment care for breast cancer survivors and their partners. The objectives of this study are to describe the development and process evaluation of a tailored informative website and to assess which characteristics of survivors and partners, participating in the feasibility study, are related to visiting the website.

**Methods:**

The development process included quantitative and qualitative assessments of survivors’ and partners’ care needs and preferences. Participants’ use and evaluation of the website were explored by conducting baseline and post-measurements. During the intervening 10–12 weeks 57 survivors and 28 partners were granted access to the website.

**Results:**

Fifty-seven percent (n=21) of survivors who took part in the post-measurement indicated that they had visited the website. Compared to non-visitors (n=16), they were more likely to have a partner and a higher income, reported higher levels of self-esteem and had completed treatment for a longer period of time. Partners who consulted the on-line information (42%, n=8) were younger and reported lower levels of social support compared to partners who did not visit the website (n=11). Visitors generally evaluated the content and lay-out positively, yet some believed the information was incomplete and impersonal.

**Conclusions:**

The website reached only about half of survivors and partners, yet was mostly well-received*.* Besides other ways of providing information and support, a website containing clear-cut and tailored information could be a useful tool in post-treatment care provision.

## Background

Increasing evidence regarding the distress and care needs of breast cancer survivors and partners after completion of primary breast cancer treatment points out the importance of adequate post-treatment care provision [1-7].

As most breast cancer patients/survivors and their partners have access to the Internet [8-10], on-line provision of psychosocial information and support shows great promise. Besides being a cost-effective and practical manner of reaching a large majority of breast cancer survivors and partners, its convenience, accessibility and anonymity make it a popular source of information [11].

Many cancer patients/survivors and their partners use the Internet to search for information and support at different stages of the illness [9,12,13]. Even after breast cancer treatment is completed, the Internet remains a major source of information [14]. As the support from hospital caregivers is largely lost after completion of breast cancer treatment [1], the re-entry phase might be a crucial moment for providing breast cancer survivors and partners with on-line information.

However, the unguided consultation of the Internet for cancer-related information has a possible downside that relates to the fact that on-line information may sometimes be conflicting or incorrect [15]. Too much and low quality on-line information could negatively impact psychological outcomes of cancer patients/survivors and their intimate partners [16,17]. Therefore, guiding survivors and partners towards reliable on-line information is of critical importance.

The Internet may offer opportunities to actively improve health care as the provision of high quality on-line information might increase breast cancer knowledge and psychosocial functioning [16,18]. However, a recent review on Internet-based information and education in the field of breast cancer stressed the need to further develop and investigate on-line provision of information and support [18].

Prerequisites for effective implementation of interventions include the systematic development of interventions [19] and careful consideration of the characteristics of the target population as well as the content and way of communicating the message [20]. Therefore, an Internet-based intervention tailored to survivors and partners during the transition phase after treatment requires a stepwise development process consisting of elaborate assessments of survivors’ and partners’ needs for information and support and their preferences regarding post-treatment care.

Another crucial step in the development of potential effective on-line tools exists of usability testing among the target population which includes assessing comprehensibility, identifying strong and weak points, and determining personal relevance [21]. Recent studies indicated that the provision of tailored on-line information by means of individualized survivorship care plans was evaluated positively by cancer survivors as well as family and friends of cancer survivors [22,23]. Users of this tool needed to answer an on-line survey regarding their demographics, cancer diagnosis and cancer treatments, which resulted in a tailored and comprehensive information package [22]. This study was set up to assess whether tailoring of on-line information to the key needs of breast cancer survivors and partners is evaluated as positively by survivors and partners as a website that is tailored to their sociodemographic and medical characteristics [22].

The present study describes the development and the process evaluation of an informative website tailored to the care needs of breast cancer survivors and partners during the transition into survivorship. Furthermore, this study intends to determine which sociodemographic, medical, and psychosocial characteristics of survivors and partners are associated with the use of the website.

### Development process of the informative website

#### Assessment of care needs and preferences regarding post-treatment care

To ensure optimal matching of the website’s content and lay-out to the needs and preferences of rehabilitating breast cancer survivors and partners, a needs assessment was conducted in a previous study among 465 survivors and 84 partners during the first 6 months after completion of primary breast cancer treatment [24]. Results indicated that survivors mainly needed information and support regarding their physical (62%) and psychological (56%) functioning, self and body image (54%), return to work (45%) and sexuality (40%) [24]. Partners’ main needs concerned the physical (54%) and psychological (52%) functioning of the survivor, how to support the survivor (49%), sexuality (33%) and the relationship with the survivor (26%).

As survivors and partners showed a strong preference for an informative website as a way of meeting their needs for information and support [24], focus group interviews were conducted to determine survivors’ and partners’ points of view regarding the look and feel of an informative website intended to support post-treatment rehabilitation. The idea of an informative website that would centralize all information of relevance during the re-entry phase appealed to survivors and partners. The website should be easy to navigate and should exude positivity. On-line interactivity (e.g. blogs, forums) was not desired, yet survivors and partners favored the possibility to download or order brochures on-line, a dictionary explaining medical terms, links to reliable websites and an overview of activities and addresses where to get support.

#### Website content and lay-out

**Figure 1:**
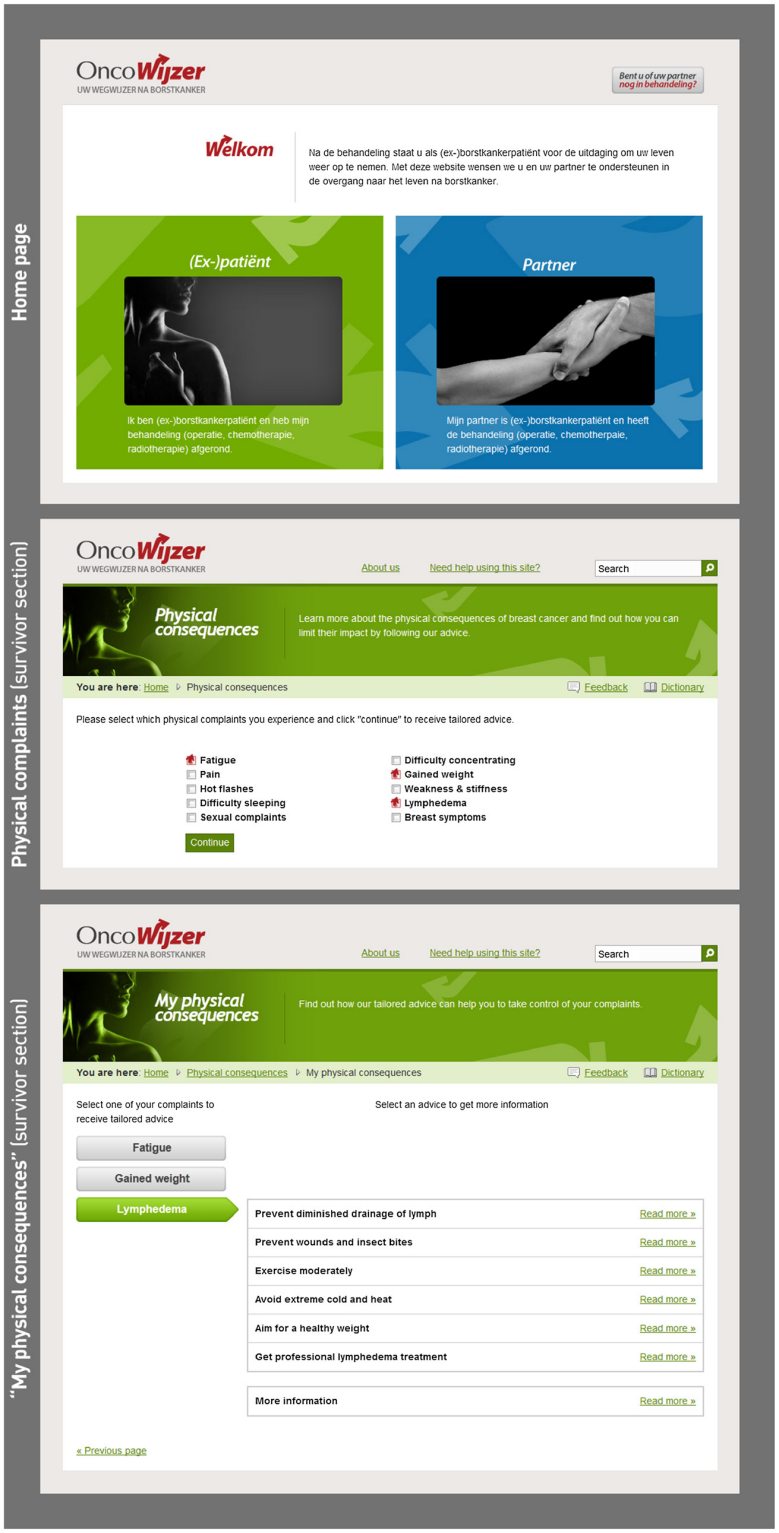
**Screenshots of the informative website.** Some parts were translated for ease of understanding.

The informative website ( http://www.oncowijzer.be) consists of two major sections, providing information for breast cancer survivors on one part and focusing on intimate partners in the other section. The topic structure of the website is displayed in Table 1. The menus and subsections of the website are based on both qualitative and quantitative research of the specific needs of survivors and partners [24]. The focus of the website on the period after breast cancer treatment is emphasized on the homepage (Figure 1). A button was provided on the homepage for people who (or whose partner) are still receiving treatment. Upon clicking on this button, links are provided towards other cancer websites.

**Table 1:** **Main menus and subsectionsof the survivor andpartner section of thewebsite**

**Survivor section**	**Partner section**
*Breast cancer*	*Breast cancer*
What is breast cancer	What is breast cancer
Research	Research
Treatment	Treatment
After treatment	After treatment
*Physical consequences*	*My complaints*
Fatigue	Overcome by emotions
Pain	Changes in the relationship
Hot flashes	Needing time for oneself
Sleeping problems	*Help guide*
Sexual complaints	Telephone
Concentration loss	E-mail
Weight gain	Open houses
Weakness / stiffness	Caregivers
Lymphedema	Internet forums
Breast symptoms	*Understanding my partner*
*Psychological consequences*	Fatigue
Fear of the future	Pain
Difficulties coping	Hot flashes
Feeling un-comprehended	Sleeping problems
Negative body image	Sexual complaints
*Social consequences*	Concentration loss
Relationship with partner	Weight gain
Starting a new relationship	Weakness / stiffness
Relationship with children	Lymphedema
Relationship with friends/family	Breast symptoms
Relationship with colleagues	Fear of the future
*Work and financial*	Difficulties coping
Return to work	Feeling un-comprehended
Financial help	Negative body image
Insurance	Return to work
Social services	*Supporting my partner*
*Life style*	Powerless and insecure
Stop smoking	Being a good listener
Sunbath safely	Supporting and not patronizing
Exercise sufficiently	
Eat healthy	
A healthy weight	
Limit the use of alcohol	
*Help guide*	
Telephone	
E-mail	
Open houses	
Volunteers	
Caregivers	
Support groups	
Internet forums	

The novelty of the website’s lay-out lies in its supply of information fitted to individual visitors’ needs. Searching for relevant topics within a profusion of information is prevented by allowing visitors to select themes that concern them from on-line lists. The choice for these topics was also based on elaborate needs assessment of the target population [24]. Depending on the menu chosen by the visitor (e.g. the *Physical consequences* menu), the menu’s subsections (e.g. fatigue, pain, hot flashes, etc.) are presented as a list in which visitors need to tick the topics about which they would like to receive more information. In Figure 1, an example is displayed of one of the website’s main menu pages on the survivor section, illustrating the possibility of selecting relevant information from a list. The content on the next page of the menu is tailored to the selection made by each visitor, providing them with a personally composed information package (e.g. merely providing information on fatigue, weight gain and lymphedema), as illustrated by Figure 1.

Another unique feature of the website is its clear-cut lay-out by drastic restriction of the number of buttons per page. The first page of the survivor section contains only 7 buttons matching the main menus displayed in Table 1, accompanied by a short description. Analogously, the first page on the partners’ section consists of only 5 buttons.

The bottom screenshot of Figure 1, displaying a tailored information package, exemplifies an additional attempt to simplify the website’s navigation. To consult the content of the personal information package, no further navigation across the website’s pages is required. Clicking on one of the complaints on the left displays advice. The advice buttons are sliding buttons, which can only be consulted one after the other. That way, the amount of information appearing at the same time on the visitors’ screen is limited.

No personal information package can be composed on the *My complaints* and *Supporting my partner* menus on the partner section of the website. These menus contain only 3 subsections, which are immediately presented as ‘sliding’ buttons on the menu page, requiring no further navigation.

Besides buttons covering the topic structure, the website contains an ‘about us’ button that provides information about the research team that developed the website and about the website’s goal. An ‘instructions for use’ button, containing directions on how to navigate the website, was added for people who are not acquainted with the Internet.

## Methods

### Procedure

The inclusion criteria and recruitment strategies for breast cancer survivors and intimate partners were similar to the previously conducted needs assessments [24]. Breast cancer survivors and intimate partners were recruited during the first half year (between 3 weeks and 6 months) after completion of primary treatment of non-metastatic primary breast cancer. Eligible women were Dutch-speaking and between 18 and 65 years old. Women above the age of 65 were excluded from the needs assessment study [24] as these women may attribute the adverse effects of treatment to causes other than breast cancer, such as the aging process and, as a consequence, are less inclined to seek treatment for managing cancer-related adverse effects. The differentiation and interaction between these processes were not the focus of the needs assessment study [24]. Other exclusion criteria were diagnosis of metastatic breast cancer or breast cancer recurrence, pregnancy and severe neurological or cognitive dysfunctions. Breast cancer survivors were recruited in 5 Flemish hospitals based on the hospital’s electronic patient file. Partners of breast cancer survivors were recruited indirectly by asking survivors who consented to participate whether a partner version of the questionnaire could be sent to their intimate partner. Upon consenting, participants received a questionnaire assessing their sociodemographic, medical and psychosocial characteristics, acquaintance with the Internet, as well as their level of care needs. Immediately after returning the questionnaire, participants received through the post a flyer containing brief information about the website and a personal log-in code for accessing the website. Use of the website was unlimited. At the log-in page, it was stressed each participant should use his/her own password in case both partners of an intimate couple took part in the study. An e-mail address was provided on the login page to retrieve their password if necessary. Passwords were automatically saved to prevent mistakes. Ten to twelve weeks after the baseline measure, participants received the post-questionnaire, assessing their use and evaluation of the informative website. Based on the personal log-in code of participants, the authors were able to verify that those participants who evaluated the website had actually visited it.

Approval of the study was granted by the ethical committee of the Ghent University Hospital (Registration number B67020096619) as from ethical committees of all other participating hospitals (University Hospitals Leuven, General Hospital Jessa Hasselt, General Hospital Klina Brasschaat, and General Hospital Aalst).

### Measures

#### Process evaluation

A post-questionnaire was developed to evaluate the content and lay-out of the tailored website, based on concepts commonly accepted in literature on process evaluation of computer-tailored interventions [25,26]. A first set of questions (five-point Likert scale: 1 = ‘I don’t agree at all’ to 5 = ‘I totally agree’) assessed to what extent the website was user-friendly, well built, interesting, informative, understandable, new, incomplete, irrelevant, unreliable, too extensive and confusing. Both positively and negatively formulated items were included. Part 2 of the questions (five-point Likert scale: 1 =’very negative’ to 5 = ‘very positive’) measured participants’ opinions about the website’s topics, use of colors, images, the ability to select information of relevance and links to other websites.

Participants were requested to rate the main menus of the website on a scale from 1 to 10. They were also asked whether consultation of the website had led them to download or order brochures on other websites (yes/no) or to get to know other websites about cancer (yes/no). Moreover, participants could write down remarks and suggestions for improvement of the website. Finally, participants who did not visit the website were asked to indicate why they had not consulted the on-line information.

Information was also gathered about participants’ use of the website by means of the website’s tracking system. In accordance with Ruland et al. [27] use of the website is measured by participants’ visits to the different sections of the website and by the duration of these visits. A visit to a certain section of the website implies that a participant has entered this section, regardless of any further actions within the section. The duration corresponds to the time spent in a website section. Each time a participant visited a webpage on the website the date and time when the webpage was entered were logged by the tracking system. The duration on each webpage was determined as the difference between the time it was entered and the entrance time of the successive webpage. As no registration occurred of the time when a webpage was left (e.g. when a participant closed his/her browser or decided to go to another website), the duration of the last webpage-visit could not be measured (as there is no successive webpage entrance time). Hence, it was not taken into account and no estimation was made on this subject (by e.g. averaging the time spent on the other sections). Interpretation of the time spent on a certain section is complicated by the diversity between the sections regarding the amount of information provided. Moreover, as all participants were able to compose an information package tailored to their individual needs, the amount of information (and consequently the time needed to go through the information) on the same website section will differ among participants. Finally, the website contained a ‘feedback’ function allowing visitors to provide feedback and suggestions about the website.

#### Sociodemographic and medical characteristics

At baseline, information was gathered regarding participants’ age, education, monthly net household income and employment. Medical information was collected regarding the date and type of breast cancer treatments of the survivor (surgery, chemotherapy, radiotherapy, immunotherapy, hormonal therapy).

#### Physical and psychosocial characteristics

Physical and psychosocial variables assessed at baseline included participants’ levels of anxiety (HADS) [28,29], depression (HADS) [28,29], self-esteem (RSE) [30], illness representations (IPQ-R) [31], social support (SSL-I) [32], lack of social support (SSL-D) [32] and coping strategies (CISS) [33,34]. Additionally, survivors’ physical side effects (EORTC-BR23) [35], fatigue (FACIT-fatigue) [36], body image (EORTC-BR23) [35], and future perspective (EORTC-BR23) [35] were measured. Partners answered additional questionnaires assessing their perceived stress (PSS) [37], and self-efficacy (GSES) [38]. A detailed description of the instruments is given elsewhere [39].

#### Acquaintance with the Internet

In the baseline questionnaire, participants were asked to indicate whether they were acquainted with the Internet (yes/no) and whether they had already used the Internet to search for cancer-related information (yes/no).

#### Care needs

The baseline measurement included the survivor and partner version of the care needs questionnaire [24], which assesses participants’ needs for information and support (three-point Likert scale: 1 = ‘not at all’, 2 = ‘somewhat’, 3 = ‘necessarily’) regarding several themes of relevance during reintegration. Each theme was measured using a single item. Themes of the survivor version of the questionnaire were: (1) physical functioning, (2) psychological functioning, (3) self and body image, (4) sexuality, (5) relationship with partner, (6) relationship with others, and (7) work, return to work and social security. The partner version’s themes were: (1) own physical functioning, (2) own psychological functioning, (3) physical functioning of the survivor, (4) psychological functioning of the survivor, (5) sexuality, (6) relationship with the survivor, (7) relationship with others (family, friends and colleagues), (8) relationship with companions, and (9) supporting the survivor. By summing the needs a total level of care needs can be calculated ranging from 7 to 21 (survivors) and 9 to 27 (partners), with higher scores indicating higher levels of care needs.

### Statistical analyses

Process evaluation was analyzed using descriptive statistics. Independent samples t-tests and chi-square tests were used to compare sociodemographic and medical characteristics, physical and psychosocial characteristics and care needs at baseline between survivors and partners who either or not visited the website. Moreover, comparisons were made between visitor and non-visitors in the website group regarding their acquaintance with the Internet. Chi-square values of dichotomous variables were compared to the Yates’ correction for continuity, which compensates for possible overestimation of the chi-square value for analysis with 2 dichotomous variables.

## Results

### Participants

**Figure 2:**
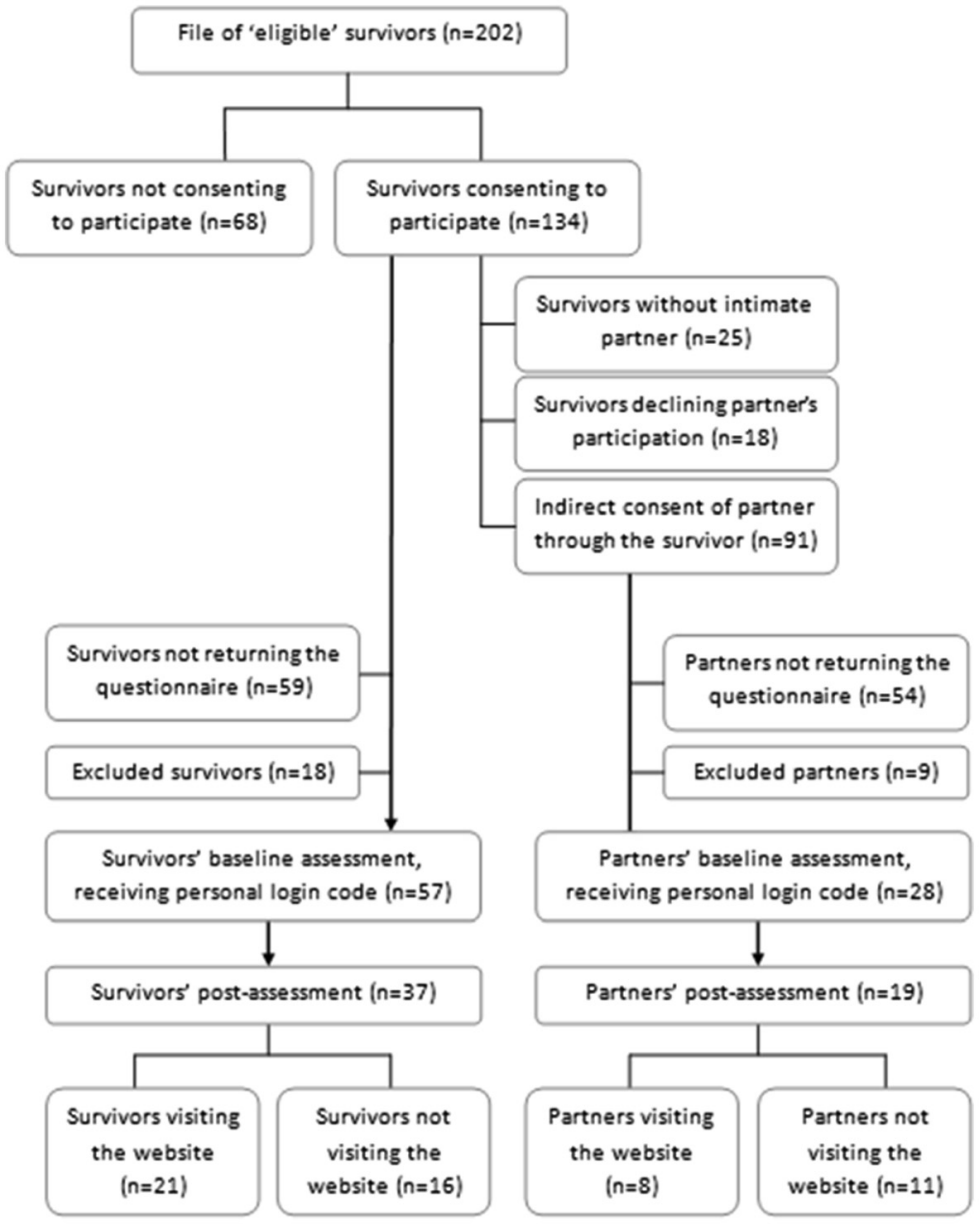
Flow-diagram of participants through the study phases.

In Figure 2 a detailed flow-chart of the recruitment process and participation of survivors and partners is presented. Nearly two-thirds (n=134) of eligible survivors (n=202) consented to participate and allowed that a questionnaire would be sent out to them. Of these survivors, 91 consented that a questionnaire would be addressed to their intimate partner. A total of 75 survivors and 37 partners returned the baseline questionnaire. After exclusion of 18 survivors and 9 intimate partners who did not meet the inclusion criteria, the effective response rate was 49.1% and 34.1% among survivors (n=57) and partners (n=28) respectively. These survivors and partners received a personal login code for accessing the website. The post-measure was answered by 37 survivors (64.9% of the baseline sample) and 19 partners (67.9% of the baseline sample). Participants’ sociodemographic and medical characteristics as well as their mean level of care needs are displayed in Table 2.

**Table 2:** Characteristics of participants

	**Survivors**		**Partners**	
	**(n=57)**		**(n=28)**	
	**%**	**M**	**%**	**M**
*Sociodemographic characteristics*				
Age		51.7		52.8
Marital state				
Partner	75.4		100	
No partner	24.6		0	
Education				
Primary/secondary school	51.8		57.7	
College graduates	48.2		42.3	
Employment				
Employed	28.1		67.9	
Unemployed/unable	71.9		32.1	
Monthly net household income				
< € 1500	22.6		4.2	
≥ € 1500	77.4		95.8	
*Medical characteristics*				
Weeks post-treat.		15.7		15.0
Surgery				
Breast conserving surgery	59.6		53.8	
Mastectomy	40.4		46.2	
Chemotherapy	71.9		73.1	
Radiotherapy	86.0		88.5	
Immunotherapy	24.6		23.1	
Hormone therapy	80.7		80.8	
*Care needs*		12.6		12.6

### Use of the website

As was registered by the tracking system, the website was visited by 21 survivors and 8 intimate partners (Figure 2). Survivors’ and partners’ total time spent on the website was on average 32 minutes and 19 minutes respectively (Table 3). The average frequency of visiting the website was 1.71 times (SD=1.10) for survivors and 1.38 times (SD=0.74) for partners.

**Table 3:** **Visits and report marksof the website menus**

	**Breast cancer survivors**				**Partners**			
	**Duration**		**Evaluation**		**Duration**		**Evaluation**	
	**n**^**a**^	**M (SD)**	**n**^**b**^	**M (SD)**	**n**^**a**^	**M (SD)**	**n**^**b**^	**M (SD)**
Entire website	21	00:32:12 (00:42:54)	–	–	8	00:18:42 (00:24:39)	–	–
Survivor section								
*Breast cancer*	16	00:13:57 (00:13:33)	20	7.53 (1.31)	–	–	–	–
*Physical consequences*	12	00:13:50 (00:17:01)	14	7.31 (0.95)	–	–		–
*Psychological consequences*	6	00:03:07 (00:03:17)	12	7.09 (1.22)	–	–		–
*Social consequences*	4	00:03:10 (00:04:28)	12	7.00 (1.18)	–	–		–
*Work and financial*	8	00:01:28 (00:01:31)	13	7.41 (1.07)	–	–		–
*Life Style*	11	00:07:23 (00:10:11)	13	7.42 (1.16)	2	00:01:06 (00:01:01)		–
*Help guide*	7	00:03:41 (00:06:27)	13	7.50 (1.09)	–	–	–	–
Partner section								
*Breast cancer*	2	00:00:07 (00:00:03)	–	–	5	00:04:54 (00:03:55)	7	6.50 (1.22)
*My complaints*	2	00:00:19 (00:00:08)	–	–	4	00:03:08 (00:03:51)	3	4.00 (1.41)
*Help guide*	1	00:00:09 ( −− )	–	–	2	00:00:59 (00:01:16)	5	6.75 (1.50)
*Understanding my partner*	2	00:00:41 (00:00:03)	–	--–	5	00:08:17 (00:13:46)	6	6.40 (1.14)
*Supporting my partner*	2	00:00:23 (00:00:16)	–	–	4	00:13:39 (00:26:05)	6	7.00 (1.87)

Duration = hours : minutes : seconds, Evaluation = report mark (/10).

n^a^ = number of participants that visited the website (menu), n^b^ = number of participants that evaluated the website (menu).

M (SD)= mean (standard deviation).

On the survivor part of the website, the *Breast cancer* and *Physical consequences* menus were visited most frequently and for the longest amount of time, viz. nearly 14 minutes (Table 3). The *Psychological* and *Social consequences* menus were consulted least and for about 3 minutes. Not all visitors of the website’s main menus visited its subsections. Per main menu, the subsection that was visited most was ‘After treatment’ (n=13) on the *Breast cancer* menu, ‘Hot flashes’ (n=6) on the *Physical consequences* menu, ‘Difficulties coping’ (n=3) on the *Psychological consequences* menu, ‘Relationship with partner’ (n=2) on the *Social consequences* menu, ‘Financial help’ (n=5) on the *Work and financial* menu, ‘A healthy weight’ (n=6) on the *Life style* menu, and ‘Caregivers’ (n=3) on the *Help guide*. A minority of survivors visited the partner section of the website. As indicated by their mean duration, these visits merely consisted of a brief scanning of the sections on the partner section.

Menus on the partner section most frequently visited by partners were the *Breast cancer* and the *Understanding my partner* menus, which were consulted for about 5 minutes and 8 minutes respectively (Table 3). On average most time was spent by partners on the *Supporting my partner* menu, viz. nearly 14 minutes. The subsection of each main menu that was visited most was ‘After treatment’ (n=3) on the *Breast cancer* menu, ‘Sexual complaints’ (n=3) on the *Understanding my partner* menu, and ‘E-mail’ (n=1), ‘Internet forum’ (n=1), and ‘Telephone’ (n=1) on the *Help guide*. As no navigation is required to consult the subsections on the *My complaints* and *Supporting my partner* menus, consultation of their subsections could not be registered. Finally, two partners had visited the *Life style* section on the survivor part of the website.

### Evaluation of the website

As shown in Table 3, the number of participants that evaluated the website sections at the post-measurement might differ from the number of participants that visited the website, as registered by the tracking system (registration of use from date of baseline assessment until date of post-measurement). Likely, some participants spread the answering of the post-measurement over several days. Coming across the questions regarding the website might have led some participants to still consult and evaluate the website, yet their visits were no longer registered.

The mean score attributed by survivors to the main menus (score between from 0 to 10) amounted to 7 or more (Table 3). Menus on the partner section were generally evaluated positively, apart from the *My complaints* menu (4.00).

On average the entire website’s content and lay-out were rated positively (Table 4). Generally participants believed the website was user-friendly, well built, interesting, informative, understandable and new. In general participants did not judge the website as being incomplete, irrelevant, implausible, too extensive and confusing. The classification of themes, the use of colors, the images, the ability to select personally relevant information and the links to websites and brochures were generally rated positively by participants. Visiting the website had led 2 survivors (9%) and 2 partners (25%) to download or order brochures on others websites. Four partners (50%) and 7 survivors (32%) indicated that they got to know other cancer websites as a consequence of visiting the website. Only one survivor consulted the ‘instructions for use’ section, which might indicate that the lay-out of the website is self-evident.

**Table 4:** **Evaluation of the contentand lay-out of thewebsite**

	**Survivors (n=22)**	**Partners (n=8)**
	**M (SD)**	**M (SD)**
Part 1		
*Positively formulated items*		
User-friendly	4.52 (0.60)	3.71 (0.95)
Well built	4.35 (0.81)	3.86 (0.69)
Interesting	3.95 (0.97)	4.00 (0.58)
Informative	3.95 (0.97)	3.14 (0.90)
Understandable	4.48 (0.60)	3.71 (0.95)
New	3.61 (1.14)	3.71 (0.76)
*Negatively formulated items*		
Incomplete	2.43 (1.12)	2.57 (0.98)
Irrelevant	1.89 (0.81)	1.71 (0.49)
Implausible	1.43 (0.51)	1.14 (0.38)
Too extensive	1.81 (0.68)	2.00 (1.41)
Confusing	1.71 (0.72)	1.86 (1.07)
Part 2		
Classification of themes	3.85 (0.81)	3.71 (0.76)
Use of colors	3.70 (0.80)	3.42 (0.79)
Images	3.75 (0.64)	3.33 (0.52)
Selection of personal information package	3.79 (0.79)	3.71 (0.76)
Links to websites and brochures	3.90 (0.85)	3.67 (0.52)

Twelve survivors (54%) and 4 partners (50%) answered the open question about their remarks and suggestions for improvement of the website. Several participants used this opportunity to share their positive experiences with the website (e.g. conveniently arranged, easy to use, interesting range of subjects, etc.). Participants’ remarks considered the fact that the information provided on the website was impersonal, vague and not new. Some suggestions were made regarding additional topics to be included in the website: e.g. more detailed information regarding survival rates according to the tumor type, catheters, side effects of medication, new medication and the course of follow-up consultations. Two survivors used of the feedback function of the website to suggest topics to be discussed in more depth: breast cancer recurrence and causes of breast cancer.

### Differences between visitors and non-visitors of the website

Sixteen survivors (43% of the survivors of the post-measure, n=37) and 11 partners (58% of the partners of the post-measure, n=19) who did not visit the website, were asked to indicate why they did not consult the on-line information. Although the course of the study was explained to participants at recruitment, 5 survivors and 3 partners reported not to have visited the website because they are not acquainted with the Internet. Eight survivors and 4 partners reported that they were not interested or did not desire any cancer-related information. Two partners reported not to have had time to visit the website. One partner forgot to visit the website and one survivor reported problems with the Internet prevented her from consulting the on-line information.

Results of the analyses of the differences between visitors and non-visitors of the website must be interpreted cautiously given the small sample sizes of survivors and partners. Compared to survivors who did not visit the website, survivors who consulted the website were more likely to have an intimate partner (χ^2^ = 5.63, p ≤ 0.05) and to fall in the higher earnings category (χ^2^ = 5.59, p ≤ 0.05). Survivors who visited the website had completed primary treatment for a longer period of time compared to survivors who did not visit the website (t= −2.23, p ≤ 0.05). Both groups generally did not differ regarding their physical and psychosocial functioning. No differences were found between visitors’ and non-visitors’ levels of anxiety, depression, illness representations, social support, lack of social support, coping strategies, body image, future perspective, physical side effects and fatigue. The only psychosocial variable that differed among both groups was survivors’ self-esteem (RSE). Survivors who visited the website reported higher levels of self-esteem (t= −3.16, p ≤ 0.01). No differences were found concerning survivors’ care needs or acquaintance with the Internet.

On average partners who visited the website were younger (48.4 years old) than their counterparts who chose not to consult the on-line information (58.4 years old) (t= 2.62, p ≤ 0.05). No differences were found concerning the medical characteristics of the partners’ spouse. Partners who visited the website reported lower levels of social support (SSL-I) (t= 2.98, p ≤ 0.01). Yet no other differences were found regarding partners’ psychosocial characteristics: anxiety, depression, self-esteem, illness representations, lack of social support, coping strategies, perceived stress and self-efficacy. Analogous to survivors, no differences were found regarding partners’ care needs or acquaintance with the Internet.

## Discussion

This study discusses the development and the process evaluation of an informative website for supporting breast cancer survivors and partners during the re-entry phase into ‘normal’ life shortly after completion of primary treatment. Quantitative and qualitative assessments of needs and preferences regarding post-treatment care [24] were used for optimal tailoring of the content of the information as well as the way it is delivered to the target population’s wishes.

The informative website distinguishes itself from existing cancer-related websites by its explicit focus on the transition period after breast cancer and on both survivors and intimate partners. A clear-cut structure was aimed for to enhance easy retrieval of information and to prevent feelings of being flooded by information. The latter was avoided further by allowing visitors to select information tailored to their needs, as the subsections on most of the website’s main menus were presented as a list in which visitors could tick themes they would like to receive more information about. As a consequence, the content of the next page on each menu was personally composed by each visitor, providing him/her with a tailored information package. By allowing each visitor to select him/herself what information he/she needs the website differs from classic ‘computer-tailoring’, in which a computerized ‘expert system’ generates the personally relevant information based on visitors’ characteristics [40]. For example, in another recently developed cancer information website visitors needed to complete a survey (regarding their demographics, cancer diagnosis and cancer treatment) which resulted in the generation of information that is specific to their situation [22]. As medical characteristics are generally unrelated to survivors’ and partners’ care needs and given the irrelevance of sociodemographic characteristics regarding partners’ needs, these characteristics may not make up a solid basis for predicting care needs shortly after completion of primary treatment [24]. Previous research did find that profiles of survivors characterized by high physical and psychological distress tend to report higher levels of needs and that partners’ emotional illness representations and negative perception of the duration of the condition of their spouse are associated with their care needs [39]. However, gathering information about these characteristics will not permit to determine what specific type of information (e.g. regarding fatigue, weight gain, lymphedema, etc.) a visitor of the website requires. Given each individual’s unique needs and experiences during the transition period, with regard to the present website a different approach was used by allowing each visitor to select him/herself what type of information he/she desires at that particular moment.

A previous needs assessment study revealed that of those survivors reporting care needs, 16 to 24 percent (depending on the rehabilitation topic in question) desired information and support by means of an informative website [24]. Partners’ preference for receiving information and support through an informative website was even more pronounced, as 22 to 37% of partners in need desired this type of care provision. In line with the fact that on-line care provision is not desired by all survivors and partners, not all participants consulted the new website. Although participants agreed to the course of the study in advance (participation in baseline and post-questionnaires with access to the website during the intervening 10 to 12 weeks) and received information about the website and a personal login code after completion of the baseline measurement, only 21 survivors (57%) and 8 partners (42%) actually consulted the website. Not visiting the website was explained by a lack of interest in cancer-related information at that moment, a lack of time or a not being acquainted with the Internet. The latter argument was not corroborated by analyses as visitors and non-visitors did not differ regarding their acquaintance with the Internet nor regarding their previous experiences in searching for on-line cancer-related information. The actual exposure of the on-line information to the target population did not measure up to its theoretical potential, given the fact that a large majority of survivors and partners have access to the Internet [8-10]. Other ways of receiving information and support, preferred by survivors and partners (e.g. informative brochures, consults with a psychologist, information sessions, etc.) may be favored by non-visitors and may constitute an essential part of post-treatment care provision [24].

Differences between participants who visited the informative website and those who did not could help identify eligible candidates for future use of the website. Previous research indicated that cancer patients’/survivors’ use of the Internet for cancer-related information and their participation in Internet-based cancer support groups is associated with aspects of a high socio-economic status, such as income, education and employment [8,11,23,41]. In the present study, survivors who visited the website were more likely to have a higher household income, yet this did not apply for their partners. Contrary to expectations, survivors’ nor partners’ education and employment status were associated with visiting the website. Compared to non-visitors of the website, larger proportions of survivors who visited the website had an intimate partner. Only among partners was younger age associated with using the website. This association was also expected to hold for survivors [8,11], but was not confirmed by the results of the present study.

Research comparing different phases of the illness continuum found that the frequency of searching the Internet for cancer-related information is lower during survivorship compared to the diagnosis and treatment phase. Nevertheless, 71% of cancer survivors still searches the Internet at least several times a year [8]. The present study’s focus on the highly specific survivorship stage shortly after treatment revealed that survivors are more inclined to consult on-line cancer-related information when treatment is completed for a longer amount a time. As a matter of fact, visitors of the website had ended treatment about 4 months ago, whereas non-visitors were on average 3 months post-treatment. It is perhaps not unlikely that the informative website is relevant to survivors and partners at later stages of survivorship. Future research ought to assess the most suitable phases of the post-treatment trajectory for on-line provision information and support.

In line with studies on the determinants of participation in Internet-based support groups use of the informative website was generally not associated with physical and psychosocial characteristics [41,42]. The only significant associations found in the present study were those between visiting the website and higher self esteem and lower levels of social support of respectively survivors and partners. In contrast to literature on women who were newly diagnosed with breast cancer [43], survivors’ nor partners’ care needs were associated with visiting the website. Given the small sample sizes of the present study, results need to be confirmed by future studies. Moreover, future research should not only assess the use of an informative website according to survivors’ and partners’ characteristics, it should also be investigated whether the effectiveness of on-line provision of cancer-related information differs according to these characteristics, taking into account participants’ physical and psychosocial profiles.

Survivors and partners who did visit the website generally evaluated its content and lay-out positively. The *Breast cancer* menu was the menu visited by the highest number of participants on the survivor section as well as the partner section (together with *Understanding my partner*). Compared to the other subsections on the *Breast cancer* menu (‘What is breast cancer’, ‘Research’, ‘Treatment’), the ‘After treatment’ subsection was visited most. This subsection discussed the following themes: ‘Control and follow-up’, ‘Reconstructive surgery’, ‘External prosthesis’ and ‘cure?’ Visitors’ interest in these themes is in line with the fact that survivors as well as partners in the previous focus group interviews mentioned that they would like these topics to be included on an informative website. One should, however, take into account that the *Breast cancer* menu is the first menu on the survivor and partner section and therefore it might be the most obvious menu to consult in discovering the new website. The positive evaluation by partners of the *Supporting my partner* menu is not surprising, considering the fact that it deals with one of the highest unmet needs reported by intimate partners. The needs assessment conducted prior to the development of the website, revealed that only 2.4% of partners who needed more information and support on how to support their spouse, stated that this need had totally been met.

The focus group interviews indicated that survivors and partners desired to retrieve addresses where to find help. In accordance to these results, in the present study the *Help guide* menu was highly valued by survivors and partners. The *My complaints* menu was judged rather negatively by partners. In the needs assessment a relatively small proportion of partners needed information regarding their own physical (16.7%) and psychological functioning (20.3%). Although this topic may not be of paramount importance to the majority of partners, the needs assessment revealed that those partners in need for such information, report that these needs are highly unmet. Therefore, for some partners, inclusion of this topic on the website might be an added value. However, interpretation of visitors’ rating of the different menus is equivocal as it is unclear whether survivors’ and partners’ scores reflect an evaluation of the quality of the menu content (whether the information is detailed enough, understandable, complete etc.) or an evaluation of its relevance to participants (partly reflecting their care needs).

The website was generally rated as interesting, informative and relevant to participants. Nonetheless, several survivors and partners made a remark concerning the impersonal and vague nature of the on-line information. The ability to compose an information package tailored to their personal situation seemed not to suffice in making all visitors feel as if the website implied a personal approach. These feelings are not unexpected considering the fact that even tailored interventions, that produce highly individualized feedback [25], are considered as ‘applying to me specifically’ by only about half of the users [44,45]. Nevertheless, some of the information on the website ought to be revised in order to make it less general and impersonal. For example, visitors desired more specific information regarding their (spouse’s) own prognosis and chances of recurrence. The information on the website regarding recurrence was limited to the average survival rate of breast cancer survivors and factors influencing the chance of recurrence (cancer stage at diagnosis, tumor size, etc.). In particular, the accompanying statements on the website that percentages of survival cannot be translated to each individual (yet ought to be discussed with the attending physician) might have led visitors to judge the information as unsatisfactory and impersonal.

Some limitations of this study should be acknowledged. Given the small sample sizes caution is called for regarding the representativeness and generalization of results. As no characteristics of non-responders were obtained, nor reasons for not participating in the questionnaire study, selection bias cannot be ruled out. One can, for example, not rule out that participants were more acquainted with the Internet compared to those who chose not to participate. Nor can one assure that the participants’ psychological well-being is representative of the target population. Furthermore, one might hypothesize that the large number of questionnaires led some survivors and partners not to participate. Moreover, given the limited statistical power characteristic of analysis within small samples sizes, one cannot rule out that visitors and non-visitors might differ more from one another than was revealed in this study.

## Conclusions

This study signifies the first step in evaluating a new informative and tailored website for supporting survivors and intimate partners during the transition period after completion of treatment. This study assessed visitors’ experiences with and evaluations of its content and lay-out and reported preliminary results about what kind of survivors and partners are more prone to consult the on-line information. As only about half of participants consulted the on-line information, the informative website as an intervention method proved not to appeal to all participants. To effectively reach breast cancer survivors and partners after completion of treatment, an informative website ought to be supplemented by other ways of providing post-treatment care (e.g. informative brochures, consults with a psychologist etc.). On-line provision of information and support ought to be considered as only one part of a multi-modal stepped-care approach. Of those who visited the website, some believed the on-line information was incomplete and impersonal. Therefore, the website’s content needs to be optimized further by adding, if possible, more detailed information regarding some topics. Nevertheless, the informative website’s content and lay-out were generally rated positively.

## Competing interests

The authors declare that they have no competing interests.

## Authors’ contributions

EP developed the website, contributed to the design of the study, performed data collection and statistical analyses, interpreted the results and drafted the manuscript. EVH, CC, LL and IDB contributed to the design of the website and the study, the interpretation of the results and the revisions of the manuscript. All authors read and approved the final manuscript.
